# Aberrant peripheral blood CD34^+^ subsets as a candidate cellular biomarker for type 2 diabetes mellitus

**DOI:** 10.1016/j.plabm.2026.e00548

**Published:** 2026-07-08

**Authors:** Itsuko Miyazawa, Junko Okano, Natsuko Ohashi, Kazunori Fujino, Miwako Katagi, Shinji Kume, Hideto Kojima

**Affiliations:** a Department of Medicine, Shiga University of Medical Science, Japan; b Department of Plastic and Reconstructive Surgery, Shiga University of Medical Science, Japan; c Department of Critical and Intensive Care Medicine, Shiga University of Medical Science, Japan; d Department of Biocommunication Development, Shiga University of Medical Science, Japan

**Keywords:** CD106^+^ TNFα^+^ proinsulin^+^ CD34^+^ subset, Cellular biomarker, Diabetic stem cell, HbA1c, Bone marrow, Type 2 diabetes mellitus

## Abstract

HbA1c and blood glucose (BS) are routinely used in diabetic care, but they do not provide direct information about ongoing cellular pathogenic processes. Previous studies in diabetic animal models identified a CD106^+^TNFα^+^proinsulin^+^ subset within short-term hematopoietic stem/progenitor cell populations, termed diabetes stem cells, that was implicated in diabetic pathology. In this study, we examined peripheral blood from patients with type 2 diabetes mellitus (T2DM) (n = 12) and healthy volunteers (n = 10). The proportion of CD106^+^TNFα^+^proinsulin^+^CD34^+^ cells was significantly higher in patients with T2DM than in healthy controls and showed a significant positive correlation with HbA1c levels. These findings suggest that this aberrant peripheral blood CD34^+^ subset may represent a candidate cellular biomarker associated with T2DM and chronic glycemic status. Further studies in larger cohorts are required to evaluate its clinical utility.

## Highlights

• Peripheral CD34^+^ subsets were profiled in patients with T2DM.
• A CD106^+^TNFα^+^proinsulin^+^CD34^+^ subset was enriched in T2DM.
• This subset correlated with HbA1c, but not blood glucose.
• Findings support this subset as an exploratory candidate cellular biomarker.

## 1 Introduction

Type 2 diabetes mellitus (T2DM) is a systemic disease characterized by insulin resistance and impaired insulin secretion [1]. Simultaneously, T2DM is accompanied by chronic low-grade inflammation and systemic metabolic stress [2]. Although various pathological conditions such as obesity, hypertension, and lack of exercise are involved in the onset of diabetes, the reasons why T2DM is so difficult to cure remain unclear.

Using animal models, we have elucidated the pathogenesis of diabetes, through which we discovered an abnormal CD106^+^ TNFα^+^ proinsulin^+^ fraction in short-term hematopoietic stem cells (ST-HSCs) in the bone marrow [3]. Their progeny migrate into the circulation and cause diabetic complications such as neuropathy, hyperphagia, and steatohepatitis [[4], [5], [6]]. These abnormal ST-HSCs do not disappear from the bone marrow by blood glucose control alone. Recently, we demonstrated that the elimination of such ST-HSCs using a histone deacetylase (HDAC) inhibitor under appropriate blood glucose control successfully led to the reset of epigenetic abnormalities in the ST-HSCs, followed by complete remission in a type 2 diabetes model in mice [7]. Therefore, we named them diabetes stem cells (DSCs).

On the basis of previous animal studies identifying a CD106^+^TNFα^+^proinsulin^+^ cell population in diabetic pathology, we hypothesized that a phenotypically related CD34^+^ cell subset may be detectable in the peripheral blood of patients with T2DM. Therefore, this exploratory study examined the association between CD106^+^TNFα^+^proinsulin^+^CD34^+^ cells and glycemic markers in patients with T2DM.

## 2 Methods

### 2.1 Participants

Twelve individuals with T2DM and ten healthy volunteers were recruited. The criteria of recruitment in this study were as follows: i) patients with diabetes whose HbA1c were 6.5%, greater than 6.5% or under treatment specific for diabetes at Shiga University of Medical Science from October 1, 2018 to October 30, 2021, ii) healthy volunteers whose HbA1c were less than 6.5% with a casual plasma glucose level <140 mg/dl or who had never experienced diabetic treatment. Diabetic complications were defined as follows: polyneuropathy, a decrease in nerve conduction velocity; retinopathy, the presence of pigment extravasation on fluorescein fundus examination; nephropathy, the presence of albuminuria; and macrovascular disorders, a history of cerebrovascular or ischemic cardiovascular disease. Informed consent was obtained by all participants and ethical approval was granted. This protocol was conducted in accordance with the principles of the Declaration of Helsinki and was approved by the Scientific Ethics Committee of Shiga University of Medical Science (R2018-102) and registered with the UMIN Clinical Trials Registry (number UMIN 000044169).

### 2.2 Flow cytometry

Flow cytometry was performed as previously described with slight modifications (Katagi 2016). Briefly, mononuclear cells were isolated using Ficoll-Paque Plus (Cytiva, Washington DC, USA) and stained with the LIVE/DEAD Fixable Blue Dead cell stain kit (Invitrogen/Thermo Fisher Scientific, MA, USA) to deplete dead cells. Next, cells were fixed with 4% paraformaldehyde and stained with the following antibodies; Human/mouse proinsulin Alexa Fluor® 488-conjugated antibody (1:200; R&D systems, Minneapolis, MN, USA), APC-conjugated anti-human CD34 (8G12) (1:20; BD Biosciences, San Jose, CA USA), Human TNFα-PE conjugated antibody (1:20; R&D systems, Minneapolis, MN, USA) and Human CD106 PE-Cy7-conjugated antibody (1:20; BioLegend, San Diego, CA, USA). Isotype-matched control antibodies were used to obtain the threshold values and determine the gate for each antibody.

### 2.3 Statistical analysis

Statistical analyses of the background of patients and healthy volunteers were performed using two-tailed t-tests. The statistical analyses on the flow cytometry data were performed using Python (version 3.11) with the SciPy statistical package and also confirmed with SPSS (version 31; IBM Corp., Armonk, NY, USA). Continuous variables are presented as mean ± standard deviation (SD). Outliers deemed physiologically implausible were interpreted as measurement errors. Consequently, they were excluded using the interquartile range (IQR) method. Specifically, the first quartile (Q1) and third quartile (Q3) were calculated for each dataset, and the IQR was defined as Q3 − Q1. Values smaller than Q1 − 1.5 × IQR or larger than Q3 + 1.5 × IQR were considered statistical outliers. After outlier removal, Spearman's rank correlation coefficient was used, and group comparisons were conducted using the Mann–Whitney *U* test. A two-tailed p-value <0.05 was considered statistically significant.

## 3 Results

A concise summary of patients with diabetes and volunteers is summarized in Table 1.

**Table 1:** Characteristics of the study subjects.

		Total	Healthy volunteers	Diabetec patinets	*t*-test (HV vs DM)
*Characteristics*					
	Number (F, M)	22 (5, 17)	10 (3, 7)	12 (2, 10)	—
	Age (years)	54.0 ± 11.6	49.0 ± 4.6	58.0 ± 14.1	ns
	Height (cm)	167 ± 7	167 ± 7	166 ± 7	ns
	Body weight (kg)	—	—	74.0 ± 14.9	—
	BMI	—	—	27.0 ± 5.3	—
	Blood glucose level (mg/dl)	111 ± 36	95 ± 20	124 ± 41	<0.05
	HbA1c (%)	8.0 ± 3.5	5.0 ± 0.14	11.0 ± 2.7	<0.01
	Duration of diabetes (years)	—	—	11.0 ± 11.6	—
	Total cholesterol (mg/dl)	197.0 ± 43.7	217.0 ± 4.6	172 ± 46.0	<0.05
	HDL cholesterol (mg/dl)	57.0 ± 17.9	73.0 ± 12.3	44.0 ± 8.9	<0.01
	Triglycerides (mg/dl)	131.0 ± 54.3	119.0 ± 50.0	140.0 ± 58.0	ns
*Complications*					
	Hypertension (%)	—	—	50	
	Dyslipidaemia (%)	—	—	75	
	Diabetic retinopathy (%)	—	—	33	
	Diabetic neuropathy (%)	—	—	33	
	Diabetic nephropathy (%)	—	—	33	
	Macroangiopathy (%)	—	—	33	
*Anti-diabetic drugs*					
	Insulin (%)	—	—	83	
	Metformin (%)	—	—	50	
	SGLT2 inhibitors (%)	—	—	42	
	DPP-4 inhibitors (%)	—	—	42	
	GLP-1 analogues (%)	—	—	42	
	Pioglitazone (%)	—	—	8	
*Other drugs*					
	Lipid-lowering agents (%)	—	—	75	
	Antiplatelet drugs (%)	—	—	25	
	Antihypertensive drugs (%)	—	—	42	

**Fig. 1:**
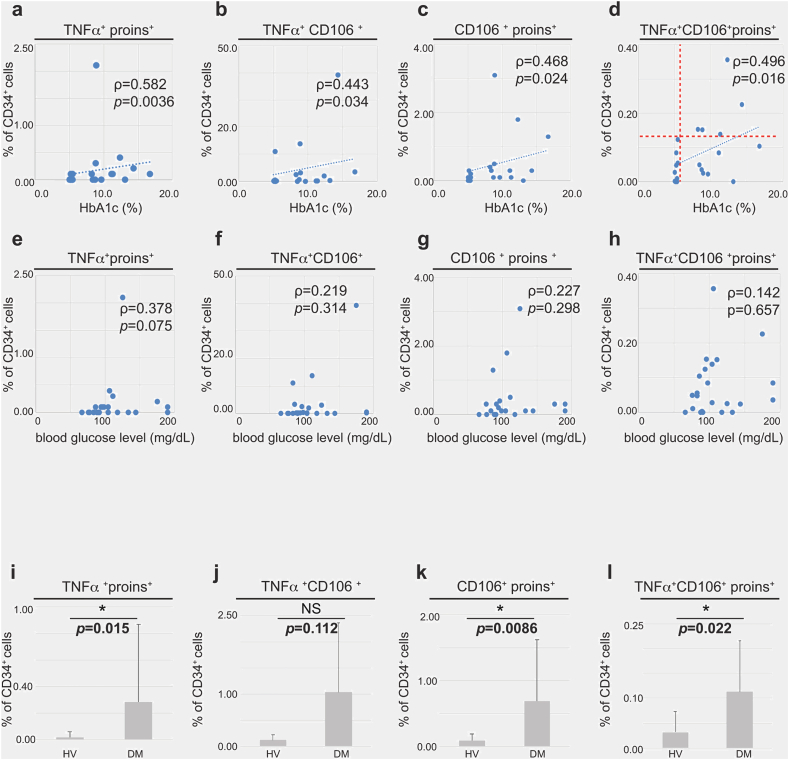
Analyses of the peripheral blood CD34^+^ subsets. (a-d) Correlations between the percentage of various subsets within CD34^+^ cells in peripheral blood and HbA1c. Threshold lines (horizontal and vertical red dot lines) in (d) were drawn from the data of the TNFα^+^ CD106^+^ proinsulin^+^ fraction within peripheral blood CD34^+^ cells and HbA1c, respectively; they were not intended to define a diagnostic cutoff. (e-h) Correlations between the percentage of various subsets in CD34^+^ cells in peripheral blood and blood glucose level. Spearman's rank correlation coefficient was used. (i-l) Mean + SD of the percentage of various subsets within CD34^+^ cells in peripheral blood in healthy volunteers (HV) and patients (DM). Comparisons between two groups were conducted using the Mann–Whitney *U* test. *p < 0.05. NS, not significant.

Flow cytometry results of patients and volunteers were collectively plotted with various combinations among TNFα^+^, proinsulin^+^, and CD106^+^ populations after gating CD34^+^ cells. The double-positive subsets within peripheral blood CD34^+^ cells—namely TNFα^+^ proinsulin^+^, TNFα^+^ CD106^+^, and CD106^+^proinsulin^+^ cells—showed significant positive correlations with HbA1c levels (Fig. 1a–c). Spearman rank correlation coefficient was ρ = 0.582, ρ = 0.443, or ρ = 0.468, while *p* values of 0.0036, 0.034, and 0.024, respectively. There was also a significant positive correlation between the percentage of the triple-positive subset, the TNFα^+^ CD106^+^ proinsulin^+^ cells of peripheral blood CD34^+^ cells and HbA1c (ρ = 0.496, *p* = 0.016) (Fig. 1d). An exploratory visualization was performed using threshold lines for HbA1c and the percentage of the TNFα^+^CD106^+^proinsulin^+^ fraction within peripheral blood CD34^+^ cells (Fig. 1d, horizontal and vertical red dotted lines). In this visualization, all healthy volunteers were below the threshold for the triple-positive subset, whereas five of twelve patients with T2DM were above it. This exploratory analysis was not intended to establish a diagnostic cutoff.

Next, the correlation of these fractions and blood glucose level was investigated. Consequently, both any two- and three-marker combinations among TNFα^+^, proinsulin^+^, CD106^+^ subsets of CD34^+^ cells did not show significant positive correlation with blood glucose level, and all *p* values exceeded 0.05 (Fig. 1e–h).

Finally, the percentages of these subsets among peripheral blood CD34^+^ cells were compared between volunteers and patients. As a result, the percentages of the TNFα^+^ proinsulin^+^ (*p* = 0.015) and CD106^+^ proinsulin^+^ (*p* = 0.0086) subsets in peripheral blood CD34^+^ cells, but not the TNFα^+^ CD106^+^ subset (*p* = 0.112), were significantly higher in patients than in volunteers (Fig. 1i–k). Of note, the percentage of the TNFα^+^ CD106^+^ proinsulin^+^ subset in peripheral blood CD34^+^ cells was significantly increased in patients compared to that in volunteers (*p* = 0.022) (Fig. 1l).

## 4 Discussion

In the present study, we identified an aberrant peripheral blood CD34^+^ cell subset, defined as CD106^+^TNFα^+^proinsulin^+^CD34^+^ cells, that was increased in patients with T2DM and positively associated with HbA1c levels. Because this was an observational and cross-sectional study, these findings do not establish whether this subset contributes causally to T2DM or diabetic complications. The results should therefore be interpreted as an exploratory association between a phenotypically defined peripheral blood CD34^+^ subset and chronic glycemic status.

Previous studies in diabetic mouse models identified a CD106^+^TNFα^+^proinsulin^+^ subpopulation within Lineage^−^Sca-1^+^c-kit^+^ cells and implicated this population in diabetic pathology [3,7]. The present human study was designed to examine whether a phenotypically related cell subset could be detected in peripheral blood from patients with T2DM. However, CD34 positivity alone does not prove that the detected cells are *bona fide* circulating hematopoietic stem cells. In addition, because proinsulin was detected at the protein level by flow cytometry, endogenous proinsulin production by these cells was not established. Non-specific antibody binding, cell contamination, or uptake of circulating proteins cannot be fully excluded. Validation using RNA-based analyses or single-cell approaches will be needed to clarify the biological identity of this subset.

HbA1c and blood glucose levels are established markers in diabetes care, but they reflect different aspects of glycemic status [[8], [9], [10]]. HbA1c reflects chronic glycemia over the preceding several weeks, whereas blood glucose levels are influenced by short-term metabolic conditions. In this study, the CD106^+^TNFα^+^proinsulin^+^CD34^+^ subset was associated with HbA1c but not with blood glucose levels, suggesting that this subset may be more closely related to chronic glycemic status than to acute glucose fluctuations. However, the present study does not establish that this subset provides independent or superior information compared with established glycemic markers.

This study has several limitations. The sample size was small, no formal a priori power calculation was performed, and the study was exploratory in nature. Therefore, the possibility of type I or type II error and the influence of outliers cannot be excluded. The observed associations should be regarded as preliminary.

In summary, we detected an aberrant CD106^+^TNFα^+^proinsulin^+^CD34^+^ subset in peripheral blood from patients with T2DM. This subset was increased in patients with T2DM and associated with HbA1c levels. These findings suggest that this peripheral blood CD34^+^ subset may represent a candidate cellular biomarker associated with T2DM-related cellular abnormalities, although its biological identity and clinical utility remain to be validated.

## Authors’ relationships and activities

I.M., J.O., N.O., M.K., K.F. and S.K. declare no conflicts of interest. H.K. is contracted as an independent advisor to Biozipcode Inc (Tokyo, Japan).

## Funding

The authors declare that financial support was received for the research, authorship, and/or publication of this article. This work is supported by a grant (PC320222) from Biozipcode, Inc.

## CRediT authorship contribution statement

**Itsuko Miyazawa:** Conceptualization, Data curation, Formal analysis, Investigation, Methodology, Writing – original draft. **Junko Okano:** Formal analysis, Supervision, Validation, Writing – original draft, Writing – review & editing. **Natsuko Ohashi:** Data curation, Methodology. **Kazunori Fujino:** Formal analysis. **Miwako Katagi:** Formal analysis. **Shinji Kume:** Conceptualization. **Hideto Kojima:** Conceptualization, Formal analysis, Supervision.

## Declaration of competing interest

H.K. is contracted as an independent advisor to Biozipcode Inc (Tokyo, Japan) and other authors declare no conflicts of interest.

## Data Availability

Data will be made available on request.
